# Effects of Nanofibrillar Nucleating Agent and Process Conditions on the Crystallization Behavior and Mechanical Properties of Isotactic Polypropylene

**DOI:** 10.3390/nano15161253

**Published:** 2025-08-14

**Authors:** Gang Wang, Mengyao Dong, Xin Pan, Xiangning Zhang, Jinlong Chen, Junfang Shen, Kun Li, Xiaoli Zhang, Jingbo Chen

**Affiliations:** 1 Key Laboratory of Material Processing and Mold Technology, School of Mechanical Engineering, Chongqing Industry Polytechnic College, Chongqing 401120, China; wanggang@cqipc.edu.cn (G.W.); dongmy@cqipc.edu.cn (M.D.); panxin@cqipc.edu.cn (X.P.); xiangningzhang@cqipc.edu.cn (X.Z.); 2 School of Materials Science and Engineering, Zhengzhou University, Zhengzhou 450001, China; zhangxl@zzu.edu.cn (X.Z.); chenjb@zzu.edu.cn (J.C.); 3 School of Intelligent Manufacturing, Luoyang Institute of Science and Technology, Luoyang 471023, China; jlchen@lit.edu.cn (J.C.); shen@lit.edu.cn (J.S.)

**Keywords:** isotactic polypropylene, nucleating agent, crystallization, mechanical properties

## Abstract

Nanofibers, as nucleating agents, can significantly alter the nucleation and growth dynamics of polymer crystallization, thereby modulating the morphology and structure of crystals to enhance mechanical performance of the materials. In this study, the effects of nanofibrillar nucleating agent 1,3:2,4-di(3,4-dimethylbenzylidene) sorbitol (DMDBS) content, melting temperature, and injection speed on the crystallization behavior and mechanical performance of isotactic polypropylene (iPP) were systematically investigated. The incorporation of DMDBS significantly increased the number of iPP nuclei, reduced crystal size and raised the onset crystallization temperature by approximately 11 °C. Concurrently, the tensile strength and elastic modulus of injection-molded iPP samples improved by 15% and 55%, respectively. However, a rise in the melting temperature led to a decrease in the crystallinity, tensile strength, elastic modulus, and impact strength of both neat iPP and iPP/DMDBS samples. With the increase in injection speed, the tensile strength and elastic modulus of iPP/DMDBS samples increased. During the crystallization process, DMDBS crystallizes prior to the iPP melt, forming the nanofibrillar network that effectively reduced the energy barrier for iPP crystal nucleation. Furthermore, under the influence of shear forces during processing, the presence of these nanofibrillar networks promoted the formation of oriented crystalline structures, which in turn contributed to the enhanced tensile strength and elastic modulus observed in iPP samples.

## 1. Introduction

The properties of semicrystalline polymers largely depend on their morphology and microstructural characteristics, which are governed by their crystallization behavior [1,2,3,4,5,6]. Isotactic polypropylene (iPP) is widely used in household products and the automotive industry due to its low density, excellent mechanical properties, good electrical insulation, resistance to corrosion, and polar solvents such as acids and alkalis, ease of processing, and low cost [7,8,9,10]. Despite these advantages, iPP also exhibits several drawbacks such as low-temperature brittleness, partial transparency, limited thermal resistance, and product shrinkage-issues that are intrinsically linked to its crystal morphology and structure. These inherent shortcomings have impeded its broader application in high-performance environments. Therefore, elucidating the relationship between the crystalline morphology and structure of iPP and its macroscopic properties is of fundamental importance for optimizing its performance and expanding its practical utility.

The crystallization behavior of iPP is primarily controlled by its nucleation process [11,12,13]. In homogeneous nucleation, thermally activated polymer segments in the iPP melt spontaneously organize into ordered chain bundles, which act as initial nuclei. This process typically yields a limited number of nuclei, prolongs crystallization time, and results in inconsistent spherulite sizes, lower crystallinity, and, ultimately, inferior mechanical properties in the final product. By contrast, heterogeneous nucleation occurs when polymer chains orderly align on the surface of “impurities” dispersed within the polymer melt [14,15,16,17]. These impurities act as nucleating agents, lowering the energy barrier for nucleation by providing ready-made nucleation sites or crystal seeds. This mechanism not only shortens the induction time but also modifies the crystal structure and morphology, thereby affecting the optical and mechanical performance of the polymer [18,19,20,21]. Additionally, nucleating agents can greatly shorten the molding cycle time, particularly in injection molding, which plays a critical role in improving production efficiency and reducing production costs [22,23,24].

Nucleating agents commonly used to enhance product performance can be classified into inorganic and organic types based on their molecular composition. Inorganic nucleating agents are mainly represented by metal oxides and hydroxides such as talc, mica, and titanium dioxide [25,26,27]. Their advantages include low cost, easy accessibility, and the ability to enhance optical clarity with minimal addition. However, their poor compatibility with iPP often leads to inadequate dispersion in the polymer matrix, thereby limiting their nucleation efficiency. Organic nucleating agents overcome the shortcomings of poor dispersion and low nucleation efficiency seen in inorganic agents, exhibiting high nucleating efficiency even at low dosages [28,29,30,31,32,33,34,35,36]. Therefore, they are the most widely used in the processing of iPP products. Among the commonly used organic nucleating agents are sorbitol and its derivatives, with the most representative being 1,3:2,4-bis(3,4-dimethylbenzylidene) sorbitol (DMDBS) [37,38,39,40,41]. Due to the presence of two free hydroxyl groups, DMDBS can form a supramolecular three-dimensional nanofibrillar network via hydrogen bonding at temperatures above the melting point of iPP. The surface of the nanofibrillar network acts as an effective heterogeneous nucleation center for iPP crystallization. Polypropylene chains adsorb onto the surface of the nanofibrillar network and undergo spatial rearrangement into ordered chain segments, which are stabilized by intermolecular interactions between DMDBS and the surrounding molten polymer chains. As a result, submicron-sized spherulites are formed, contributing to improved mechanical properties [42]. Using in situ small-angle X-ray scattering (SAXS) and electron microscopy, Lipp et al. observed the development of nanofibrillar networks during the solidification of DMDBS in molten PP [43]. Sowinski et al. reported that DMDBS facilitates the crystallization of orthorhombic γ-form iPP under high pressure, with the γ-phase content depending on DMDBS concentration [44]. Simth et al. proposed that DMDBS induces the crystallization of PP through epitaxial growth of PP chains along the DMDBS surface, which accelerates the transformation from random coils to helical structures in the PP melt and stabilizes the helical conformation [45]. In injection molding, the final properties of polymeric materials are influenced not only by the incorporation of nucleating agents such as DMDBS, but also by critical processing parameters, including melt temperature and injection speed. During processing, the polymer melt experiences substantial shear stress, which facilitates the alignment of molecular chains along the flow direction and promotes the development of oriented crystalline structures, thereby improving mechanical performance. Additionally, the partial retention of pre-existing ordered structures during melting provides favorable conditions for flow-induced crystallization under shear. The synergistic interplay between nucleating agents and processing conditions plays a pivotal role in modulating crystallization behavior and the mechanical properties of semi-crystalline polymers. However, the synergistic effects of nucleating agents and processing conditions on the crystallization behavior and mechanical properties of iPP remain insufficiently explored.

This study systematically investigates the effects of the DMDBS nucleating agent, melt temperature and injection speed on the crystallization behavior of iPP, with a particular emphasis on elucidating the relationship between crystalline morphology, structure, and macroscopic properties. The findings aim to provide fundamental insights for the rational design and development of high-performance semicrystalline polymer materials.

## 2. Experimental Section

### 2.1. Materials

A semicrystalline isotactic polypropylene (iPP, Brand No. T30S, Ningxia, China) with a weight-average molecular weight (*M*_w_) of 300 kg/mol and a dispersity (Đ) of 5.2 was used as purchased. The iPP possesses a specific gravity of 0.9 g/cm^3^, an isotacticity of ≥98.0%, and a melt flow index of 3.5 g/10 min (at 210 °C, 2.16 kg). The nucleating agent 1,3:2,4-di(3,4-dimethylbenzylidene) sorbital (DMDBS, Brand No. Millad 3988) was purchased from Milliken, Inc. (Hong Kong, China) and used without further purification, and the detailed information of the above materials was summarized in the Table S1.

### 2.2. Samples Preparation

To eliminate residual moisture, both pure iPP and DMDBS were dried separately in a vacuum oven at 80 °C for 4 h. iPP compounds containing 0.2, 0.5, and 0.7 wt% DMDBS, along with a reference sample without any additive, were first dry-blended at room temperature and subsequently melt-blended at 230 °C for 10 min using a micro twin-screw extruder (SHJ-20, Nanjing Jieente Mechanical and Electrical Co., Ltd., Nanjing, China) operating at a screw speed of 80 rpm. The processing temperature was sufficiently high to ensure the complete dissolution of DMDBS into the iPP matrix. The samples were prepared using an injection molding machine (Demag, Düsseldorf, Germany). The formulations of the DMDBS content, melting temperature, and injection molding speeds are listed in Table 1.

### 2.3. Thermal Analysis

The melting point and crystallinity of iPP and iPP/DMDBS blends were characterized using a differential scanning calorimeter (DSC-Q2000, TA Instruments, New Castle, DE, USA) under a high purity nitrogen atmosphere (See Figures S1–S3 and Tables S2–S5). High-purity indium was used as a calibration standard to ensure the accuracy and reliability of the thermal measurements. Samples weighing between 5 and 10 mg were heated from 30 to 200 °C at a constant rate of 10 °C/min under nitrogen flow. The crystallinity $\chi_{\alpha }$ of both neat iPP and iPP/DMDBS blends was calculated using the following equation:

(1) $$\chi_{\alpha }=\left(\frac{\Delta H_{\alpha }}{\Delta H_{\alpha }^{0}}\right)\times 100\%$$

$\Delta H_{\alpha }$ represents melting enthalpy for iPP. The fusion enthalpy $\Delta H_{\alpha }^{0}$ of 100% crystalline polymer is 209 J/g for iPP [46,47,48].

### 2.4. Mechanical Properties Analysis

Static tensile tests were performed in accordance with GB/T 1040-92 [49] using an Instron 5585 universal testing machine (Instron Co., Norwood, MA, USA) at room temperature. A minimum of five to seven specimens was tested under each condition, and the average values were reported. The testing speed was set at 50 mm/min.

For evaluating the impact strength variation, we fabricated an GB/T 1843-1996 [50] standard-type bar, incorporating a V-shaped groove at the center of the sample. Impact strength testing experiments were conducted using a ZBC1501 type Izod tester (MTS Industrial Systems Co., Ltd., Beijing, China).

## 3. Results and Discussion

### 3.1. The Effect of DMDBS Contents on Crystallization Behavior and Mechanical Properties of Isotactic Polypropylene

**Figure 1:**
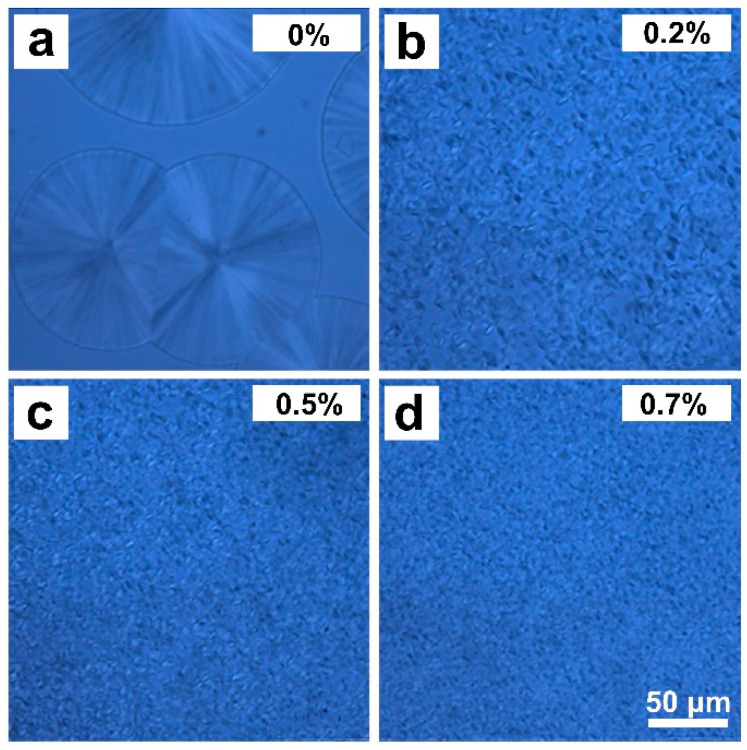
Polarized optical photomicrographs of iPP crystals obtained by isothermal crystallization at 145 °C for 1 h with different contents of DMDBS (**a**) 0 wt.%, (**b**) 0.2 wt.%, (**c**) 0.5 wt.%, and (**d**) 0.7 wt.%. The size of the scale bar is 50 μm.

Samples for microscopy were prepared by melting the pelletized polymer (prepared by using a micro twin-screw extruder) between a slide and a cover slip on a hot stage at 230 °C, followed by rapid quenching in an ice-water bath. Crystallization of iPP compounds containing different DMDBS content was initiated by heating the samples on a Linkam THMS 600 hot stage (Linkam Scientific Instruments, Tadworth, UK). The crystallization process was continuously monitored in real time with an optical microscope (Olympus BX-51, Tokyo, Japan). The protocol adopted for our crystallization experiments consisted of the following steps: (1) Heating the samples from room temperature to 230 °C at a rate of 10 °C/min; (2) holding at 230 °C for 3 min; (3) cooling at 30 °C/min down to 140 °C and holding at 140 °C for 1 h; and (4) quenching to room temperature for subsequent morphological analysis. Representative morphologies of iPP crystals with different DMDBS contents, as captured via optical microscopy, are shown in Figure 1a–d. As expected, the crystalline morphology exhibited a strong dependence on the concentration of DMDBS in the blends.

Under identical crystallization conditions, pure iPP samples exhibited relatively large crystals, limited in number and unevenly distributed. In contrast, the iPP/DMDBS blends exhibit a significantly greater number of crystals, which are smaller and more uniformly dispersed [44]. The pronounced reduction in crystal size serves as compelling evidence of the high nucleation efficiency imparted by DMDBS. In neat iPP, crystallization proceeds via homogeneous nucleation, which involves a high energy barrier. This results in the formation of fewer nuclei and allows for extensive crystal growth, yielding large spherulites. In the iPP/DMDBS system, DMDBS is fully dissolved in the polymer melt at elevated temperatures (230 °C). Upon cooling to the isothermal crystallization temperature, DMDBS self-assembles into a three-dimensional nanofibrillar network via hydrogen bonding within the iPP matrix. The high surface area of this nanostructured network provides abundant nucleation sites, significantly lowering the energy barrier and promoting widespread crystal formation through a heterogeneous nucleation mechanism. Consequently, the increased nucleation density and restricted growth space lead to a greater number of smaller crystals.

**Figure 2:**
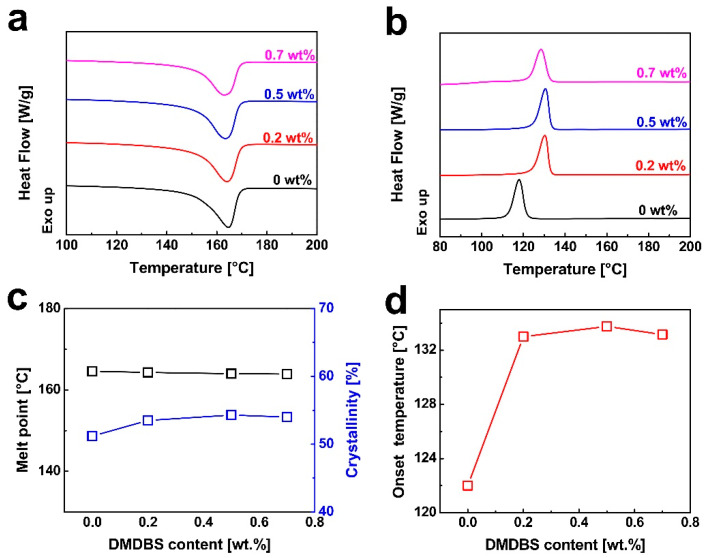
Comparison of the DSC melting curves (**a**) and cooling curves (**b**) of iPP containing different contents of DMDBS. (**c**) The evolution of the melting point and the relative crystallization fraction *X*_t_ (%) as a function of DMDBS contents. (**d**) The evolution of onset crystallization temperature as a function of DMDBS contents.

Figure 2a,b show the DSC heating (melting) and cooling (crystallization) curves of iPP samples containing various concentrations of DMDBS, respectively. Figure 2c presents the melting temperatures and crystallinities extracted from the DSC heating curves shown in Figure 2a. The melting point of iPP crystals shows minimal variation with increasing DMDBS content. This invariance may be attributed to the fact that the melting point is governed by lamellar thickness, which is dictated by the crystallization temperature. Given that crystallization was performed under identical thermal conditions, the lamellae in all samples are presumed to have similar thicknesses. Nevertheless, a modest increase in crystallinity is observed with rising DMDBS concentration, which can be attributed to the enhanced nucleation density induced by DMDBS, facilitating the formation of more crystalline regions within the matrix. For the DSC cooling analysis (Figure 2b), samples were first heated from room temperature to 230 °C at a rate of 10 °C/min, held isothermally for 3 min and subsequently cooled to 60 °C at 10 °C/min. The onset crystallization temperature of neat iPP was recorded at 122 °C. Upon the addition of 0.2 wt% DMDBS, this temperature increased significantly to 133 °C, as shown in Figure 2d, clearly indicating the strong nucleating capability of DMDBS. This enhancement is attributed to the formation of a three-dimensional nanofibrillar network by DMDBS, which effectively lowers the energy barrier for nucleation and enables crystallization to initiate at higher temperatures. The crystallization peak also shifts to higher temperatures with the DMDBS addition, further confirming its nucleation effect. However, at a DMDBS concentration of 0.7 wt%, a slight decrease in the onset and peak crystallization temperatures is observed compared to samples with 0.2 wt% and 0.5 wt% DMDBS. This reduction may be due to the aggregation of DMDBS at higher loadings, which diminishes its dispersion and thereby weakens its nucleating efficiency.

**Figure 3:**
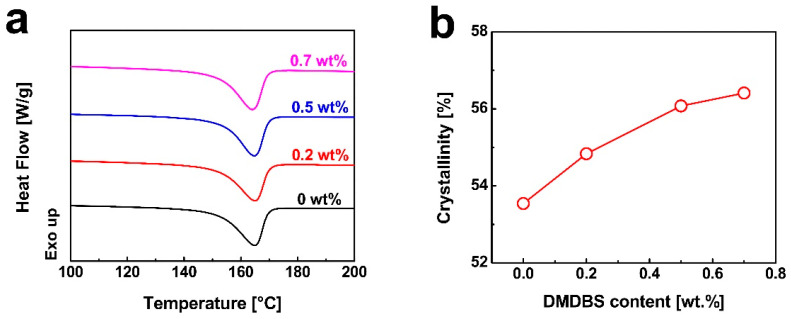
(**a**) Comparison of the DSC melting curves of iPP injection-molded samples containing different contents of DMDBS. (**b**) The evolution of the relative crystallization fraction *X*_t_ (%) of iPP injection-molded samples as a function of DMDBS contents. The iPP injection-molded samples were prepared under the following conditions: melt temperature of 210 °C, injection speed of 10 cm^3^/s, injection pressure of 80 MPa, and holding pressure of 60 MPa.

The DSC heating curves of injection-molded iPP samples are shown in Figure 3a. The processing parameters for the injection-molded iPP samples included a melt temperature of 210 °C, injection speed of 10 cm^3^/s, injection pressure of 80 MPa, and a holding pressure of 60 MPa. As shown in Figure 3a, the melting points of the samples remain nearly unchanged regardless of DMDBS content. The crystallinity values, derived from the corresponding DSC curves, are summarized in Figure 3b. A clear trend is observed in which the crystallinity of injection-molded iPP increases with rising DMDBS concentration, further corroborating the nucleating efficacy of DMDBS in promoting polymer crystallization during processing.

**Figure 4:**
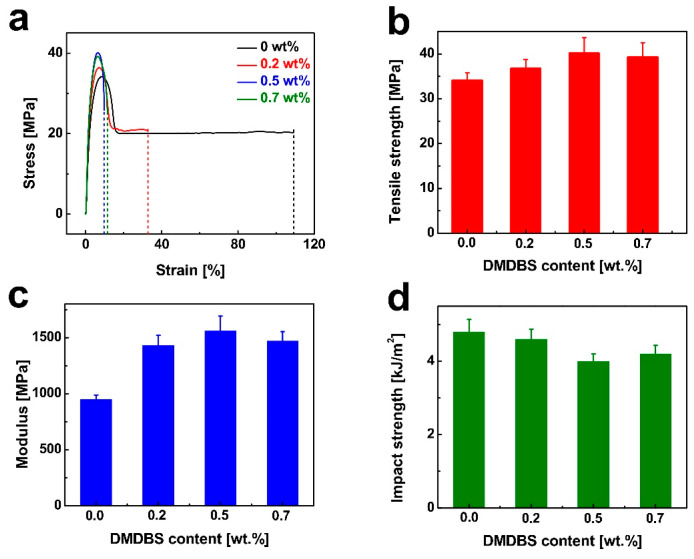
(**a**) The plots of stress for iPP injection-molded samples containing different contents of DMDBS as a function of strain. The tensile strength (**b**), modulus (**c**), and impact strength (**d**) of iPP injection-molded samples as a function of DMDBS content.

The stress–strain behavior of injection-molded iPP samples is illustrated in Figure 4a. During tensile testing, the pure iPP exhibited a typical thermoplastic deformation sequence: initial elastic deformation, yielding accompanied by necking, development of large strains, and eventual fracture. In the early stage of loading, stress increases linearly with strain in accordance with Hooke’s law. In this elastic region, deformation primarily arises from variations in bond lengths and angles within the iPP chains, and is fully reversible upon unloading. The slope of this linear segment represents the elastic modulus, which quantifies the material’s stiffness and resistance to deformation under applied stress. Following the elastic region, stress–strain behavior becomes nonlinear. As strain continues to increase, the stress gradually decreases, the cross-sectional area of the sample becomes non-uniform, and necking becomes evident. At this stage, deformation is no longer fully reversible, indicating the transition to plastic deformation. This irreversible deformation is attributed to the stretching and alignment of polymer chains in the amorphous regions, with additional contributions from lamellar slippage. In the absence of significant strain hardening, the maximum stress observed is defined as the tensile strength of the material. Upon further straining, stress remains relatively constant until final fracture. Figure 4b,c present the tensile strength and elastic modulus values extracted from the stress–strain curves. Both properties are significantly enhanced by the incorporation of DMDBS. At a DMDBS loading of 0.5 wt%, the tensile strength and elastic modulus reached maximum values of 40.1 MPa and 1558.7 MPa, representing improvements of 17.6% and 59.1%, respectively, compared to neat iPP. These enhancements can be attributed to the nucleating role of DMDBS. During processing, DMDBS precipitates earlier than iPP and forms a nanofibrillar network, which promotes extensive and uniform crystal nucleation [51]. Consequently, the crystal grains become finer, reducing internal stress concentrations. Additionally, the increased crystallinity and improved chain alignment induced by DMDBS enhance intermolecular interactions, restrict chain slippage, and improve the material’s resistance to deformation, thereby elevating both tensile strength and modulus [52]. However, it is worth noting that increased DMDBS content leads to a reduction in impact resistance. The amorphous phase plays a critical role in absorbing impact energy. As DMDBS loading increases, the crystallinity rises and the proportion of the amorphous phase decreases, thereby diminishing energy dissipation capacity and reducing impact strength.

### 3.2. The Effect of Melt Temperature on Crystallization Behavior and Mechanical Properties of Isotactic Polypropylene

**Figure 5:**
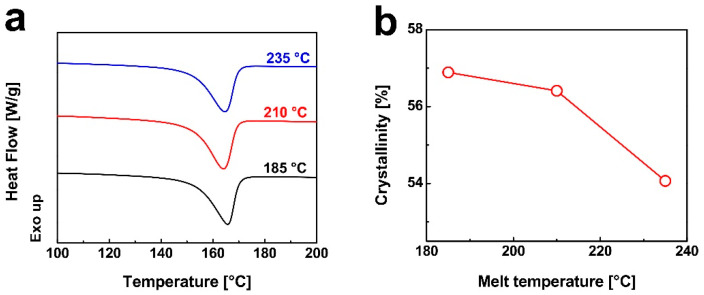
(**a**) Comparison of the DSC melting curves of iPP/0.7 wt% DMDBS injection-molded samples prepared under different melt temperature. (**b**) The evolution of the relative crystallization fraction *X*_t_ (%) of iPP/0.7 wt% DMDBS injection-molded samples as a function of melt temperature. The iPP/0.7 wt% DMDBS injection-molded samples were prepared under the following conditions: injection speed of 10 cm^3^/s, injection pressure of 80 MPa, and holding pressure of 60 MPa.

Figure 5a presents the DSC heating curves of injection-molded iPP/0.7 wt% DMDBS samples processed at various melt temperatures. The melting point remains largely unaffected by the change in melt temperature. However, as shown in Figure 5b, the crystallinity of the samples decreases progressively with increasing melt temperature. At relatively low melt temperatures, DMDBS nanofibers are not fully dissolved and, thus, partially retain their fibrous morphology. During injection molding, these residual nanofibrils undergo orientation at regions experiencing high shear and extensional flow, such as the nozzle entrance and exit. Upon subsequent cooling, these oriented nanofibrils act as effective heterogeneous nucleation sites, thereby promoting extensive iPP crystallization and yielding higher overall crystallinity. In contrast, at elevated melt temperatures, DMDBS nanofibrils are more likely to fully dissolve in the polymer melt. Although shear and extensional flow still induce alignment of DMDBS structures, the elevated temperature renders these oriented domains thermally unstable and susceptible to relaxation. Consequently, the number of aligned nanofibrils is reduced, diminishing their ability to facilitate nucleation. As a result, the nucleation efficiency of DMDBS is compromised at higher processing temperatures, leading to decreased crystallinity in the final product [53].

**Figure 6:**
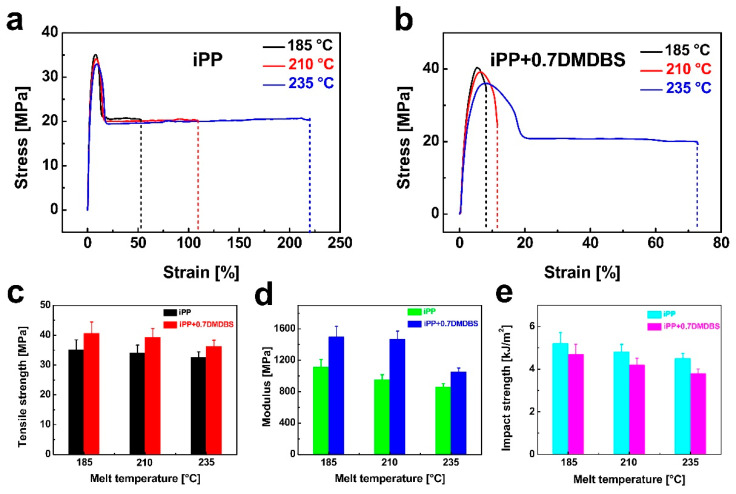
The plots of stress for iPP (**a**) and iPP/0.7 wt% DMDBS (**b**) injection-molded samples prepared under different melt temperatures as a function of strain. The tensile strength (**c**), modulus (**d**), and impact strength (**e**) of iPP and iPP/0.7 wt% DMDBS injection-molded samples as a function of melt temperature.

Figure 6a,b present the stress–strain curves of injection-molded neat iPP and iPP/0.7 wt% DMDBS samples processed at different melt temperatures. The corresponding tensile strength and elastic modulus values extracted from these curves are summarized in Figure 6c,d, respectively. For neat iPP, processing at a melt temperature of 235 °C led to decreases in tensile strength and modulus by 7.1% and 22.6%, respectively, compared to values obtained at 185 °C. In contrast, the iPP/0.7 wt% DMDBS samples showed reductions of 10.6% in tensile strength and 29.8% in modulus when processed at 235 °C versus 185 °C. These reductions can be primarily attributed to the adverse effects of elevated melt temperatures on the crystallization behavior of the polymer. Specifically, higher processing temperatures tend to reduce overall crystallinity and disrupt the formation of oriented crystalline structures, both of which are critical for enhancing the mechanical properties of semicrystalline polymers. Figure 6e illustrates the variations in impact strength of both neat iPP and iPP/0.7 wt% DMDBS samples as a function of melt temperature. For neat iPP, the impact strength decreased by 13.5% as the melt temperature increased from 185 °C to 235 °C. Similarly, iPP samples containing 0.7 wt% DMDBS exhibited a 19.1% reduction in impact strength over the same temperature range.

### 3.3. The Effect of Injection Speeds on Cell Structure and Mechanical Properties of Polypropylene–Polyethylene Composites

**Figure 7:**
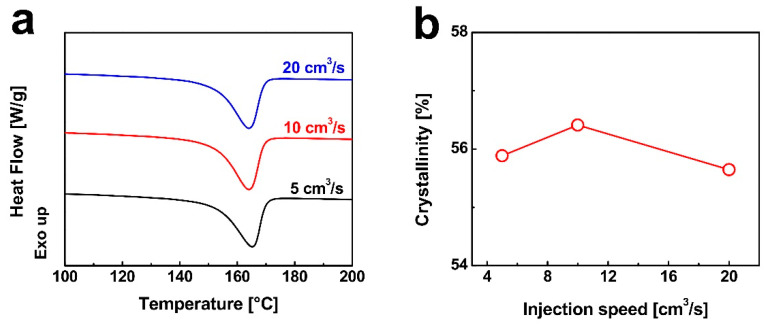
(**a**) Comparison of the DSC melting curves of iPP/0.7 wt% DMDBS injection-molded samples prepared under different injection speeds. (**b**) The evolution of the relative crystallization fraction *X*_t_ (%) of iPP/0.7 wt% DMDBS injection-molded samples as a function of injection speeds. The iPP/0.7 wt% DMDBS injection-molded samples were prepared under the following conditions: melt temperature of 210 °C, injection pressure of 80 MPa, and holding pressure of 60 MPa.

Figure 7a displays the DSC heating curves of injection-molded iPP/0.7 wt% DMDBS samples under different injection speeds, showing that the melting point remains nearly unchanged. However, with increasing injection speed, crystallinity first increases and then decreases (see Figure 7b). The shear and extensional flow in the molding process promotes polymer chains alignment along the flow direction, which aids in forming nucleation sites for crystallization. As the injection speed increased from 5 cm^3^/s to 10 cm^3^/s, the intensified shear flow significantly promoted the alignment of DMDBS nanofibrils along the flow direction, forming oriented nanofibrillar structures that provided abundant nucleation sites for iPP, thereby inducing extensive crystallization and increasing overall crystallinity. Unexpectedly, further increasing the injection speed to 20 cm^3^/s does not lead to a continued rise in crystallinity. Although the stronger shear theoretically favors the formation of more oriented DMDBS structures, it also results in increased viscous heating, elevating the local melt temperature. The elevated temperature, in turn, compromises the thermal stability of the aligned nanofibrils, promoting their relaxation and diminishing their nucleation efficiency. Consequently, the effective number of nucleation sites decreases, leading to a reduction in crystallinity at high injection speeds.

**Figure 8:**
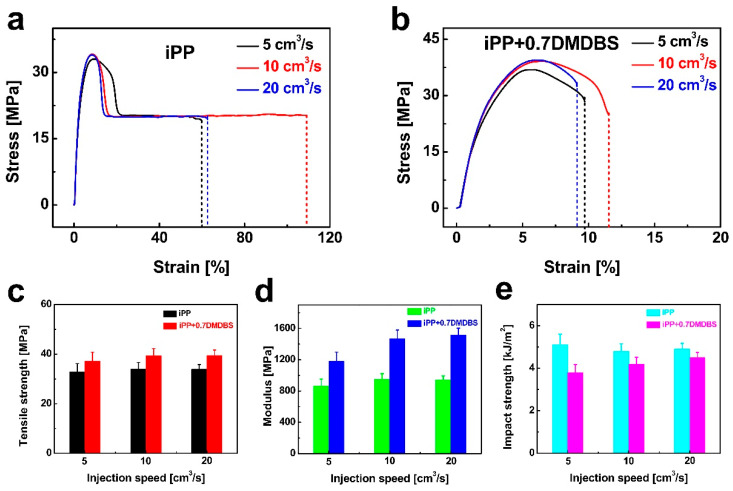
The plots of stress for iPP (**a**) and iPP/0.7 wt% DMDBS (**b**) injection-molded samples prepared under different injection speeds as a function of strain. The tensile strength (**c**), modulus (**d**), and impact strength (**e**) of iPP and iPP/0.7 wt% DMDBS injection-molded samples as a function of injection speeds.

Figure 8a,b show the stress–strain curves of injection-molded neat iPP and iPP/0.7 wt% DMDBS samples processed at different injection speeds. The corresponding tensile strength and elastic modulus values, extracted from these curves, are summarized in Figure 8c,d.

For both neat iPP and iPP/0.7 wt% DMDBS samples, an increase in injection speed led to improved tensile strength, with gains of 3.6% and 6.5%, respectively. The elastic modulus of neat iPP increased by 9.6%, while that of the DMDBS-modified samples exhibited a more substantial increase by 28.8%, nearly three times the improvement observed for pure iPP. These enhancements in tensile properties are closely associated with the formation of oriented crystalline structures and elevated crystallinity under higher shear conditions during injection molding. The presence of DMDBS nanofibrillar networks further promotes chain alignment and nucleation, contributing to superior mechanical performance. Figure 8d illustrates the variation in impact strength of iPP and iPP/0.7 wt% DMDBS samples as a function of injection speed. For neat iPP, the impact strength initially decreases and then exhibits a modest recovery at higher speeds. In contrast, the impact strength of iPP/0.7 wt% DMDBS samples increases progressively with injection speed. This improvement is likely attributable to the uniform refinement of spherulitic structures induced by DMDBS, which facilitates more homogeneous stress distribution and enhances the material’s ability to dissipate impact energy.

## 4. Conclusions

The mechanical behavior of crystalline polymers is governed not only by their chemical structure but also by the crystallization morphology and structures developed during processing. Incorporating DMDBS into iPP alters its crystalline morphology and structure, thereby tuning its mechanical properties. A comprehensive study was conducted to examine the effects of DMDBS content, melt temperature, and injection speed on the crystallization behavior and mechanical performance of iPP. DMDBS acts as an efficient heterogeneous nucleating agent, inducing a transformation in the crystallization mode of iPP from homogeneous to heterogeneous nucleation. Compared to homogeneous nucleation, heterogeneous nucleation leads to a higher number of more uniformly distributed nuclei in the polymer melt, promoting finer and more evenly dispersed crystalline grains, and thereby enhancing crystallinity. Moreover, the onset crystallization temperature of iPP increases by approximately 11 °C upon the addition of DMDBS, highlighting its strong nucleating activity. During injection molding, the shear and extensional forces generated at higher injection speeds enhance the alignment of DMDBS nanofibrils along the flow direction. This alignment facilitates the development of oriented crystalline structures, which in turn improve the tensile strength and elastic modulus. Additionally, at lower melt temperatures, DMDBS nanocrystals may not be completely melted and can become oriented during flow. Upon subsequent cooling, these pre-aligned structures act as potent nucleation sites, further accelerating crystallization. The combined effects of enhanced nucleation and flow-induced orientation lead to significant improvements in mechanical properties, including tensile strength, modulus, and impact resistance.

## Figures and Tables

**Table 1:** Value of the DMDBS content, melting temperature, and injection molding speeds.

Number	DMDBS Content	Melting Temperature (°C)	Injection Molding Speeds
1	0	210	10
2	0.2	210	10
3	0.5	210	10
4	0.7	210	10
5	0.7	185	10
6	0.7	235	10
7	0.7	210	5
8	0.7	210	20

## Data Availability

The original contributions presented in this study are included in the article/Supplementary Material. Further inquiries can be directed to the corresponding author.

## Supplementary Materials

The following supporting information can be downloaded at the following address: https://www.mdpi.com/article/10.3390/nano15161253/s1.
