# Supplementary material for: Effects of Nanofibrillar Nucleating Agent and Process Conditions on the Crystallization Behavior and Mechanical Properties of Isotactic Polypropylene
Source: Nanomaterials (Basel). 2025 Aug 14;15(16):1253. doi: 10.3390/nano15161253 (PMC12388222; doi:10.3390/nano15161253)
Supplement: Supplementary file 1 [file nanomaterials-15-01253-s001.zip › nanomaterials-3775214-supplementary.pdf]

Supporting Information for

Effects of nanofibrillar nucleating agent and process conditions on the crystallization behavior and mechanical properties of isotactic polypropylene

Gang Wang[1,2], Mengyao Dong[1], Xin Pan[1], Xiangning Zhang[1], Jinlong Chen[3], Junfang Shen[3], Kun Li[2,*], Xiaoli Zhang[2], Jingbo Chen[2]

[1]Key Laboratory of Material Processing and Mold Technology, School of Mechanical Engineering, Chongqing Industry Polytechnic College, Chongqing 401120, China

[2]School of Materials Science and Engineering, Zhengzhou University, Zhengzhou 450001, China

[3]School of Intelligent Manufacturing, Luoyang Institute of Science and Technology, Luoyang, 471023, China

Corresponding authors:

[*]*kunli@zzu.edu.cn*

1. Value of the properties of the materials used

Table S1. Value of the properties of the materials used

Materials	Brand No	M_w	Dispersity index (Đ)	Melt flow index (210 °C, 2.16 kg)	Specific gravity	isotacticity
iPP	T30S	300 kg/mol	5.2	3.5 g/10 min	0.9 g/cm^3	≥ 98.0%
DMDBS	Millad 3988	/	/	/	/	/

2. Estimation of the melting point and crystallinity by DSC

The melting point and crystallinity of neat iPP and iPP/DMDBS blends were characterized using differential scanning calorimetry (DSC-Q2000, TA Instruments, USA) under a high-purity nitrogen atmosphere. High-purity indium served as the calibration standard to ensure the accuracy of thermal measurements. Samples weighing 5-10 mg were heated from 30 °C to 200 °C at a constant rate of 10 °C/min under nitrogen flow. The crystallinity χ_α of both neat iPP and iPP/DMDBS blends was calculated using the following equation:

$$\chi_\alpha = \left(\frac{\Delta H_\alpha}{\Delta H_\alpha^0}\right)\times 100\% \quad (1)$$

ΔH_α represent melting enthalpy for iPP. The fusion enthalpy ΔH_α^0 of 100% crystalline polymer is 209 J/g for iPP.

To illustrate the procedure for analyzing the melting point and crystallinity of the samples, we used the TA Universal Analysis software provided with the DSC instrument. Sample 1, prepared with 0 wt% DMDBS at a melting temperature of 210 °C and an injection speed of 10 cm^3/ s, was selected as an example. The DSC dataset for this sample was labeled as “0-210-10.” The analysis was performed as follows:

(1) The TA Universal Analysis software was launched, and the dataset “0-210-10” was opened, displaying the corresponding melting curve (Figure S1).

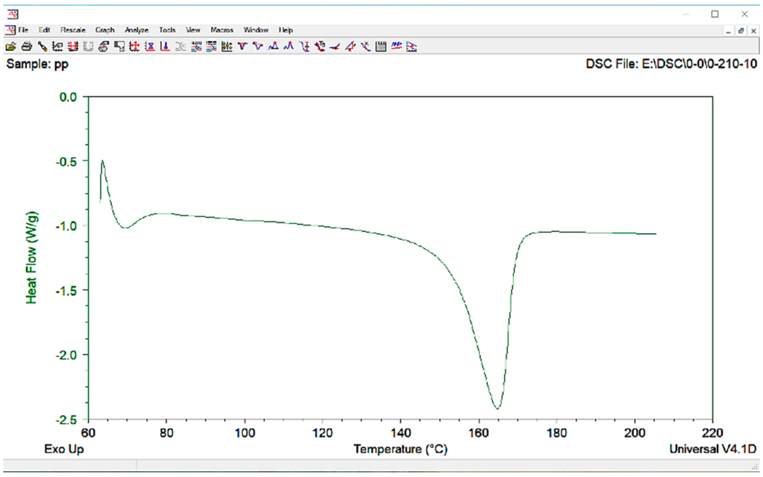

Figure S1. The DSC melting curves of iPP injection-molded samples. The iPP injection-molded samples were prepared under the following conditions: DMDBS contents of 0 wt%, melt temperature of 210 °C, injection speed of 10 cm³/s.

(2) From the main menu, Analyze > Peak Integration > Linear was selected, and two markers were placed at the start and end points of the baseline by double-clicking the curve.

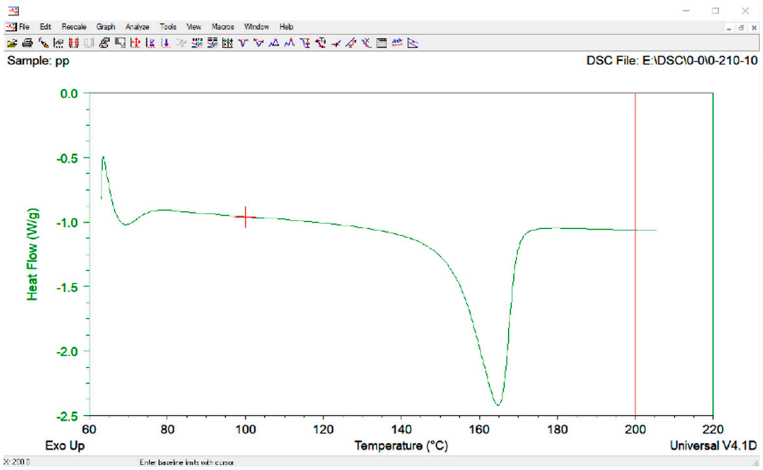

Figure S2. Markers for the integration range of the DSC melting curve of the iPP injection-molded sample.

(3) A right-click opened the analysis menu, from which Accept Limits was selected to generate the integration results.

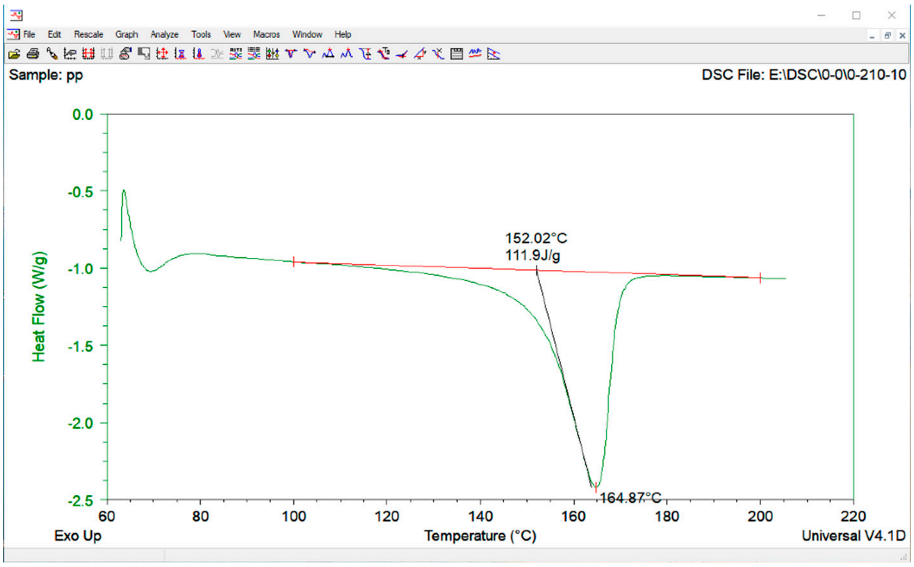

Figure S3. DSC melting curve integration results of iPP injection molded samples.

(4) As shown in Figure S3, the melting point (peak temperature) and melting enthalpy of Sample 1 were determined to be 164.87 °C and 111.9 J/g, respectively. Using Equation (1), the crystallinity was calculated to be 53.5%;

(5) The same procedure was applied to all other samples, and the calculated melting points and crystallinities are summarized in Tables S2-S5.

Table S2. Melt point T_m, melting enthalpy ΔH_α and crystallinity of samples in Figure 2 in the manuscript calculated from DSC data.

DMDBS contents	T_m (°C)	ΔH_α	χ_α (%)
0 wt.%	164.57	106.4	50.9
0.2 wt.%	164.01	111.1	53.2
0.5 wt.%	163.45	113.5	54.3
0.7 wt.%	163.91	112.8	54.0

Table S3. Melt point T_m, melting enthalpy ΔH_α and crystallinity of samples in Figure 3 calculated from DSC data.

DMDBS contents	T_m (°C)	ΔH_α	χ_α (%)
0 wt.%	164.87	111.9	53.5
0.2 wt.%	164.93	114.6	54.8
0.5 wt.%	164.75	117.2	56.1
0.7 wt.%	164.06	117.9	56.4

Table S4. Melt point T_m, melting enthalpy ΔH_α and crystallinity of samples in Figure 5 calculated from DSC data.

Melt temperature (°C)	T_m (°C)	ΔH_α	χ_α (%)
185	165.67	118.9	56.9
210	164.06	117.9	56.4
235	164.60	113.1	54.1

Table S5. Melt point T_m, melting enthalpy ΔH_α and crystallinity of samples in Figure 7 calculated from DSC data.

Injection speeds (cm^3/s)	T_m (°C)	ΔH_α	χ_α (%)
5	165.26	116.8	56.9
10	164.06	117.9	56.4
20	164.03	114.2	54.1
